# Long-term mortality after isolated coronary artery bypass grafting and risk factors for mortality

**DOI:** 10.1186/s13019-024-02943-0

**Published:** 2024-07-10

**Authors:** Therese K.T. Chua, Fei Gao, Shaw Yang Chia, Kenny Y.K. Sin, Madhava J. Naik, Teing Ee Tan, Yi Chuan Tham

**Affiliations:** 1 Lee Kong Chian School of Medicine, Nanyang Technological University, 11 Mandalay Road, Singapore, 308232 Singapore; 2 National Heart Centre Singapore, Singapore, Singapore; 3 Duke-NUS Medical School, Singapore, Singapore; 4 Singapore Cardiac Data Bank, National Heart Centre Singapore, Singapore, Singapore; 5 Cardiothoracic Surgery Department, National Heart Centre Singapore, Singapore, Singapore

**Keywords:** Coronary artery bypass grafting, Mortality, Risk factors

## Abstract

**Background:**

Patients requiring coronary artery bypass grafting (CABG) have multiple co-morbidities which need to be considered in totality when determining surgical risks. The objective of this study is to evaluate short-term and long-term mortality rates of CABG surgery, as well as to identify the most significant risk factors for mortality after isolated CABG.

**Methods:**

All patients with complete dataset who underwent isolated CABG between January 2008 and December 2017 were included. Univariate and multivariate Cox regression was performed to determine the risk factors for all-cause mortality. Classification and regression tree analysis was performed to identify the relative importance of these risk factors.

**Results:**

3,573 patients were included in the study. Overall mortality rate was 25.7%. In-hospital mortality rate was 1.62% overall. 30-day, 1-year, 5-year, 10-year and 14.5-year mortality rates were 1.46%, 2.94%, 9.89%, 22.79% and 36.30% respectively. Factors associated with death after adjustment for other risk factors were older age, lower body mass index (BMI), hypertension, diabetes mellitus, chronic obstructive pulmonary disease, pre-operative renal failure on dialysis, higher last pre-operative creatinine level, lower estimated glomerular filtration rate (eGFR), heart failure, lower left ventricular ejection fraction and New York Heart Association class II, III and IV. Additionally, female gender and logistic EuroSCORE were associated with death on univariate Cox analysis, but not associated with death after adjustment with multivariate Cox analysis. Using CART analysis, the strongest predictor of mortality was pre-operative eGFR < 46.9, followed by logistic EuroSCORE *≥* 2.4.

**Conclusion:**

Poorer renal function, quantified by a lower eGFR, is the best predictor of post-CABG mortality. Amongst other risk factors, logistic EuroSCORE, age, diabetes and BMI had a relatively greater impact on mortality. Patients with chronic kidney disease stage 3B and above are at highest risk for mortality. We hope these findings heighten awareness to optimise current medical therapy in preserving renal function upon diagnosis of any atherosclerotic disease and risk factors contributing to coronary artery disease.

**Supplementary Information:**

The online version contains supplementary material available at 10.1186/s13019-024-02943-0.

## Introduction

Coronary artery bypass grafting (CABG) is the most common cardiac surgery performed, and one of the most common major surgeries done worldwide. It is a cornerstone in the management of coronary artery disease (CAD). Even with the advancement in minimally invasive percutaneous coronary intervention (PCI), several large studies have shown that CABG still has superior benefits, especially in more extensive coronary disease [1, 2]. As patients requiring CABG often have multiple co-morbidities, extensive investigation into the factors affecting mortality of patients undergoing the procedure has been done. Some widely recognized risk factors include older age, female gender, poorer renal function, and the presence of diabetes mellitus [3, 4]. The development of risk algorithms has also been useful in predicting the risk of mortality when considering all the risk factors in totality. Several widely adopted models include the EuroSCORE and the Society of Thoracic Surgeons (STS) score [5–7]. This information helps to best select patients who would benefit from CABG intervention, and thus aid in the clinical decision to proceed with a surgical management.

There is a high burden of CAD in the Singapore population, with ischemic heart disease being one of the top three causes of mortality, accounting for 19.7% of all deaths in the country [8]. As such, there is a need to continuously review CABG surgery outcomes and the factors affecting them to guide local clinical practice. The objective of this study is to evaluate short-term and long-term mortality rates of CABG surgery, as well as to identify the most significant risk factors for mortality after isolated CABG. We performed further subgroup analysis for gender as the mortality rate diverged very early after isolated CABG.

## Methods

This study is a retrospective case-note and database review of all patients who had undergone isolated CABG procedures between January 2008 and December 2017 at the National Heart Centre Singapore (NHCS), a tertiary cardiac surgery referral center in Singapore. Ethical approval was obtained from Singhealth Centralised Institutional Review Board (CIRB 2022/2150).

Comprehensive data collection at baseline such as demographics, medical history and risk factors, procedural data, medications, complications, and discharge outcomes were captured in our database. Trained coordinators use a standardized case report form to collect data and entered these findings into an electronic database, which underwent internal and external validation. The primary outcome measured was all-cause mortality and all these outcomes through September 2022 were obtained from hospital records, supplemented by data from the National Registry of Death.

### Statistical analysis

Continuous variables are expressed as median (IQR), and categorical variables as n (%). For comparisons between men and women, Mann–Whitney test was performed for continuous variables, and Chi-square test for categorical variables. To determine the risk factors influencing long-term all-cause mortality, univariate Cox regression was performed. Subsequently, risk factors that were significant in the univariate regression (*p* < 0.05) were included in the multivariate Cox regression. The cumulative survival was plotted using the Kaplan-Meier method. All statistical analyses were performed using STATA 18 (College Station, Texas, USA). For all analyses, a two-tailed P-value of < 0.05 was considered significant. Classification and regression tree (CART) analysis was performed on all risk factors for mortality to identify the optimal categorization that discriminates mortality outcomes.

## Results

**Fig. 1:**
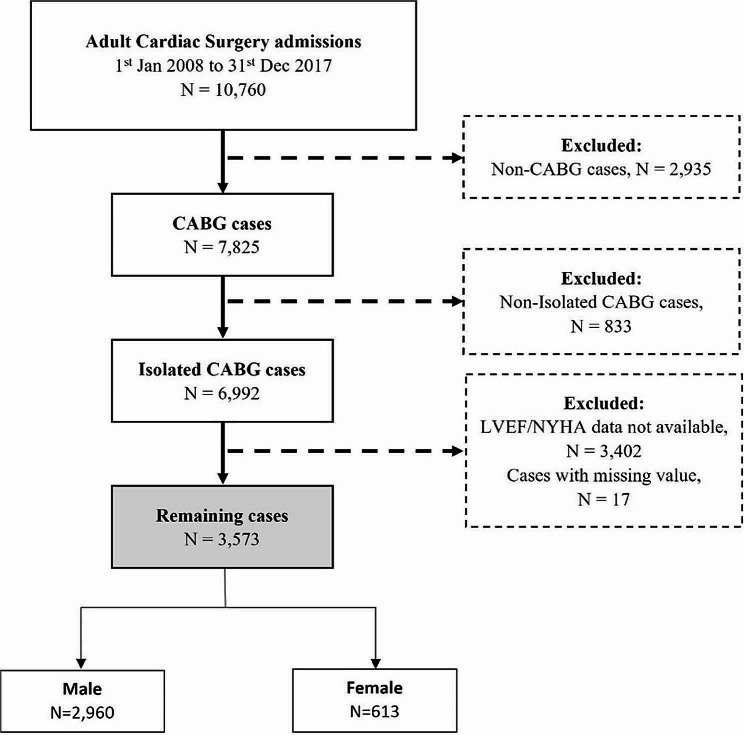
Flowchart of the selection process of study population. Final study population consisted of patients who underwent isolated CABG with complete data available

Between 1 January 2008 and 31 December 2017, 6,992 patients who underwent isolated CABG at NHCS were retrospectively identified. Of these, 3,419 patients were excluded due to missing data. The final population analyzed in this study consisted of 3,573 patients (male *n* = 2,960 [82.8%], female *n* = 613 [17.2%]) (Fig. 1). The minimum period of follow-up was 5 years and the maximum period was 14.7 years.

Background characteristics of the population are shown in Table 1. The median age was 62 years (IQR 55–68), with a higher median age in females than males (65 years vs. 61 years; *p* < 0.001). The most common co-morbidities amongst the study population were dyslipidemia (82.8%), hypertension (75.4%) and diabetes mellitus (51.7%). The female population had significantly higher incidence of diabetes mellitus (66.9 vs. 48.5%, *p* < 0.001), hypertension (83.7 vs. 73.7%, *p* < 0.001) and heart failure (8.0 vs. 5.7%, *p* = 0.035). They also had lower estimated glomerular filtration rate (eGFR) (61.6 vs. 73.5mL/min/1.73m^2^, *p* < 0.001) and higher logistic EuroSCORE (2.9 vs. 1.8, *p* < 0.001) compared to the males. The male population had higher last pre-operative creatinine levels (89.0 vs. 70.5µmol/L, *p* < 0.001) and lower left ventricular ejection fraction (LVEF) (53 vs. 55%, *p* < 0.001) than the females.

**Table 1:** Background characteristics of study population. Continuous variables are expressed as median (IQR) and categorical variables as n (%)

Patient Characteristics	Overall (*N* = 3,573)	Female (*N* = 613)	Male (*N* = 2,960)	*p*-value
**Age (years)**	62 (55–68)	65 (60–71)	61 (55–67)	< 0.001
**Body mass index (kg/m** ^**2**^ **)**	25.2 (22.9–27.8)	25.4 (22.8–28.4)	25.1 (22.9–27.6)	0.140
**Diabetes mellitus**	1,846 (51.7%)	410 (66.9%)	1,436 (48.5%)	< 0.001
**Hypertension**	2,694 (75.4%)	513 (83.7%)	2,181 (73.7%)	< 0.001
**Dyslipidemia**	2,958 (82.8%)	522 (85.2%)	2,436 (82.3%)	0.088
**eGFR (mL/min/1.73m** ^**2**^ **)**	71.4 (54.9–91.1)	61.6 (44.6–82.3)	73.5 (57.1–92.8)	< 0.001
**Last pre-operative creatinine level (µmol/L)**	88 (74–105)	70.5 (60–97)	89 (78–106)	< 0.001
**Pre-operative renal failure on dialysis**				
**No**	3,419 (95.7%)	574 (93.6%)	2,845 (96.1%)	
**Yes**	154 (4.3%)	39 (6.4%)	115 (3.9%)	
**COPD**	34 (1.0%)	4 (0.7%)	30 (1.0%)	0.400
**Heart failure**	219 (6.1%)	49 (8.0%)	170 (5.7%)	0.035
**LVEF (%)**	53 (40–60)	55 (45–61)	53 (40–60)	< 0.001
**NYHA**				
**I**	299 (8.4%)	39 (6.4%)	260 (8.8%)	
**II**	2,299 (64.3%)	390 (63.6%)	1,909 (64.5%)	
**III**	868 (24.3%)	162 (26.4%)	706 (23.9%)	
**IV**	107 (3.0%)	22 (3.6%)	85 (2.9%)	
**Logistic EuroSCORE**	2.1 (1.3–3.3)	2.9 (1.9–4.4)	1.8 (1.3–3.1)	< 0.001

Over the total duration of study, 917 patients reached the endpoint of all-cause death (mortality rate 25.7%). This consisted of 199 females (mortality rate 32.5%) and 718 males (mortality rate 24.3%). In terms of number of all-cause deaths per person years, the overall death rate was 26.86 per 1,000 person years (95% CI 25.18–28.65). Females had a death rate of 35.70 per 1,000 person years (95% CI 31.06–41.04), whereas males had a death rate of 25.13 per 1,000 person years (95% CI 23.36–27.04) (Table 2).

**Table 2:** Mortality rate of all-cause death in patients who underwent isolated CABG

	Number of all-cause deaths	Mortality rate	Person-years	Rate per 1,000 person-years	95% CI
**Overall**	917	25.7%	34,141.57	26.86	25.18–28.65
**Female**	199	32.5%	5,574.97	35.70	31.06–41.04
**Male**	718	24.3%	28,566.60	25.13	23.36–27.04

In-hospital mortality rate was 1.62% overall (female 3.26%; male 1.28%). This was defined as death occurring within the same hospital admission that CABG was done. When stratified into specific time intervals, the overall mortality rates at 30 days, 1 year, 5 years, 10 years and 14.5-years were 1.5%, 2.9%, 9.9%, 22.8% and 36.3% respectively. In females, the 30-day, 1-year, 5-year, 10-year and 14.5-year mortality rates were 2.6%, 5.9%, 14.5%, 30.0% and 44.0% respectively. In males, the 30-day, 1-year, 5-year, 10-year and 14.5-year mortality rates were 1.2%, 2.3%, 8.9%, 21.3% and 34.6% respectively (Table 3).

**Table 3:** Short-term and long-term mortality rates of patients who underwent isolated CABG

	Time	At risk	Fail	Mortality rate
**Overall**	In-hospital	3,573	58	1.6%
	30 days	3,521	53	1.5%
	1 year	3,468	248	2.9%
	5 years	3,161	365	9.9%
	10 years	1,957	191	22.8%
	14.5 year	82	2	36.3%
**Female**	In-hospital	613	20	3.3%
	30 days	597	20	2.6%
	1 year	577	53	5.9%
	5 years	517	76	14.5%
	10 years	309	33	30.0%
	14.5 year	14	0	44.0%
**Male**	In-hospital	2,960	38	1.3%
	30 days	2,924	33	1.2%
	1 year	2,891	195	2.3%
	5 years	2,644	289	8.9%
	10 years	1,648	158	21.3%
	14.5 year	68	2	34.6%

The results of the Cox proportional hazard analysis for risk factors associated with mortality after isolated CABG are shown in Table 4. Based on univariate Cox analysis, the risk factors associated with death were older age, female gender, lower body mass index (BMI), hypertension, diabetes mellitus, chronic obstructive pulmonary disease (COPD), pre-operative renal failure on dialysis, higher last pre-operative creatinine level, lower eGFR, higher logistic EuroSCORE, heart failure, lower LVEF and New York Heart Association (NYHA) class II, III and IV. After adjustment with multivariate analysis, female gender and higher logistic EuroSCORE were not associated with mortality. All other risk factors for mortality on univariate analysis remained risk factors after adjustment.

**Table 4:** Univariate and multivariate analysis of risk factors for post-CABG mortality using the Cox proportional hazard model

Risk factors	Hazard ratio (95% CI)	*p*-value	Adjusted hazard ratio (95% CI)	*p*-value
**Age (years)**	1.06 (1.06–1.07)	< 0.001	1.04 (1.03–1.05)	< 0.001
**BMI (kg/m** ^**2**^ **)**	0.96 (0.94–0.98)	< 0.001	1.03 (1.01–1.05)	0.008
**Female gender**	1.42 (1.22–1.67)	< 0.001	1.01 (0.86–1.20)	0.880
**Diabetes mellitus**	2.03 (1.77–2.33)	< 0.001	1.71 (1.48–1.97)	< 0.001
**Hypertension**	1.79 (1.50–2.12)	< 0.001	1.33 (1.11–1.60)	0.002
**Dyslipidemia**	1.16 (0.97–1.39)	0.096		
**eGFR (mL/min/1.73m** ^**2**^ **)**	0.972 (0.97–0.98)	< 0.001	0.986 (0.98–0.99)	< 0.001
**Last pre-operative creatinine level (µmol/L)**	1.0022 (1.00–1.00)	< 0.001	1.001 (1.00–1.00)	0.002
**Pre-operative renal failure on dialysis**				
**No**	1		1	
**Yes**	4.23 (3.46–5.19)	< 0.001	1.52 (1.17–1.98)	0.002
**COPD**	2.41 (1.47–3.96)	< 0.001	1.98 (1.18–3.33)	0.009
**Heart failure**	2.51 (2.03–3.11)	< 0.001	1.26 (1.00-1.58)	0.047
**LVEF**	0.97 (0.97–0.98)	< 0.001	0.978 (0.97–0.98)	< 0.001
**NYHA**				
**I**	1		1	
**II**	1.95 (1.29–2.93)	0.001	1.85 (1.23–2.79)	0.003
**III**	2.44 (1.60–3.71)	< 0.001	1.87 (1.23–2.86)	0.004
**IV**	3.5 (2.11–5.81)	< 0.001	2.26 (1.35–3.80)	0.002
**Logistic EuroSCORE**	1.08 (1.07–1.09)	< 0.001	1.01 (1.00-1.02)	0.081

**Fig. 2:**
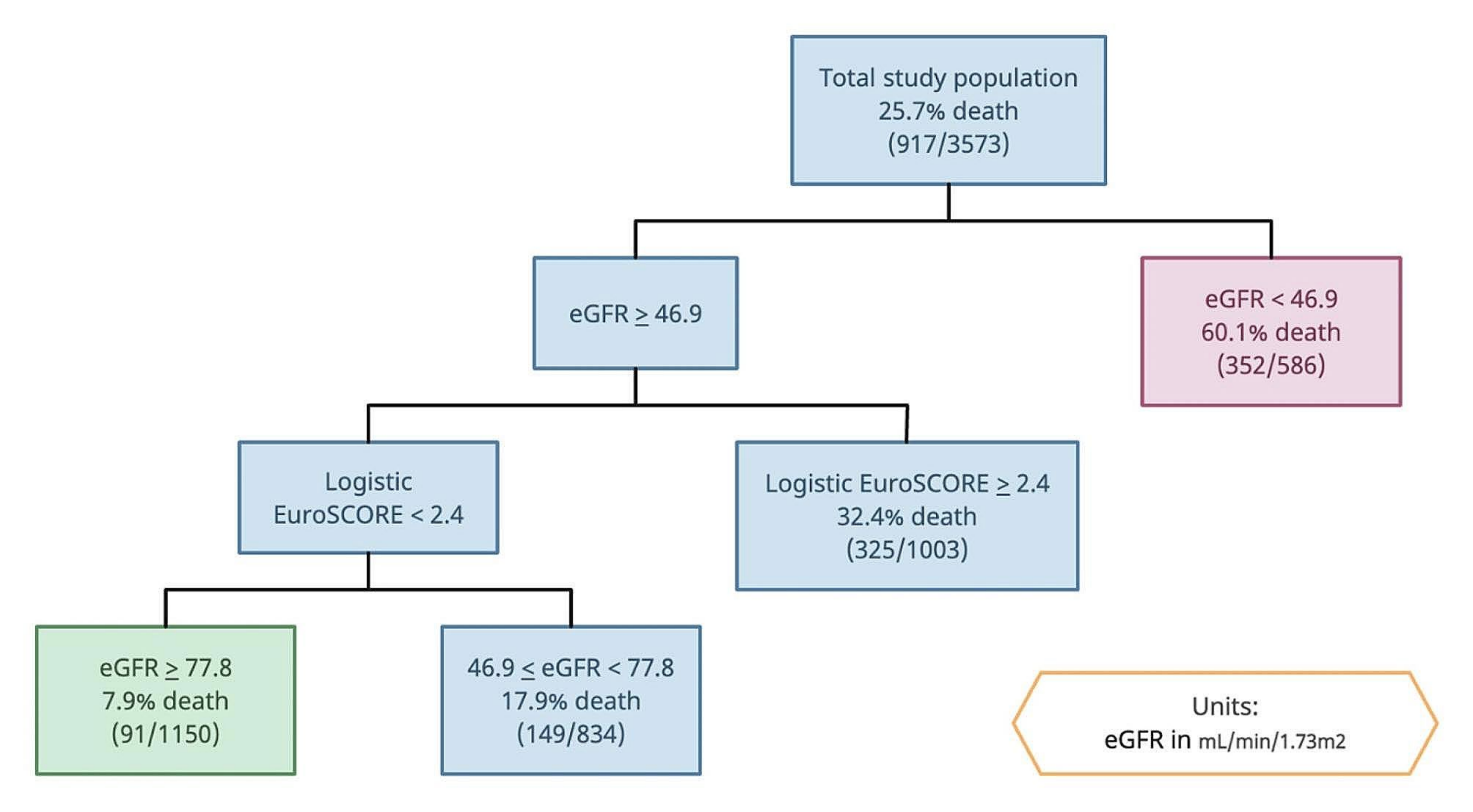
CART analysis for mortality after CABG. Variables branching off to the right are associated with higher mortality. Variables branching off to the left are associated with lower mortality. Green: combination with the best prognosis. Red: combination with the worst prognosis

**Fig. 3:**
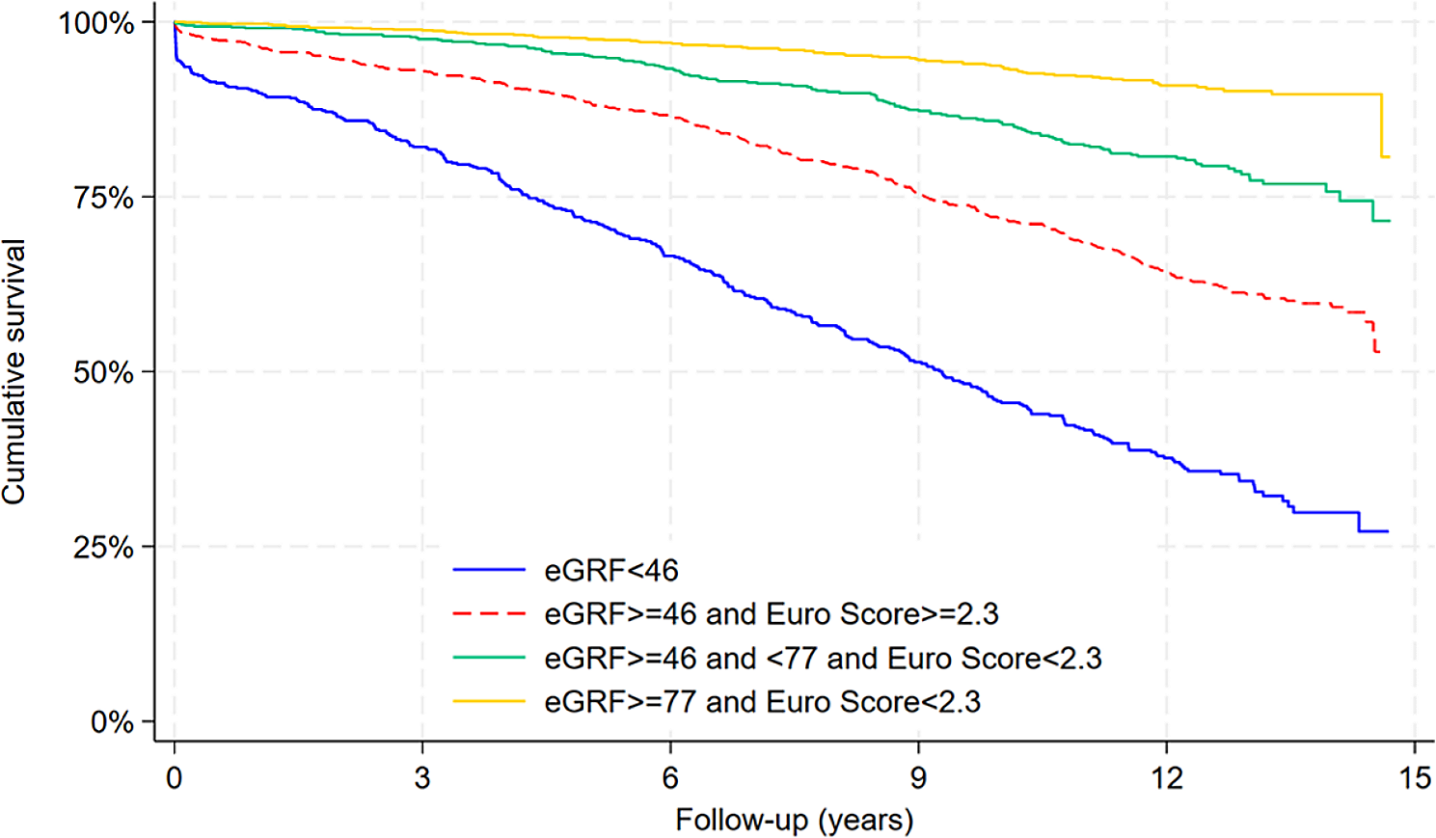
Kaplan-Meier survival curve for risk factors that are the strongest predictors of mortality after CABG

CART analysis for the overall population is shown in Fig. 2. Parameters branching off to the right of the tree are associated with higher mortality compared to those branching to the left. The strongest predictor of all-cause death after isolated CABG was pre-operative eGFR. Patients with eGFR < 46.9mL/min/1.73m^2^ had a 60.1% death rate. In patients with eGFR *≥* 46.9mL/min/1.73m^2^, logistic EuroSCORE was the next most important predictor of death. Those with a logistic EuroSCORE of *≥* 2.4 had a death rate of 32.4%. In patients with a logistic EuroSCORE < 2.4, those with 46.9 *≤* eGFR < 77.8mL/min/1.73m^2^ had a death rate of 17.9%. In patients with a logistic EuroSCORE < 2.4, those with eGFR *≥* 77.8mL/min/1.73m^2^ had a death rate of 7.9% (Fig. 2). Figure 3 shows the Kaplan-Meier survival curves for these four groups of patients.

For CART analysis specific to female patients, the strongest predictor of death was eGFR (cut-off < 36.7mL/min/1.73m^2^). In patients with lower eGFR, the next most important predictor of death was last pre-operative creatinine *≥* 133.5µmol/L. In patients with higher eGFR, the next discriminant was last pre-operative creatinine *≥* 76.5µmol/L. In those with better eGFR but worse last pre-operative creatinine levels, logistic EuroSCORE (cut-off *≥* 3.35) was the next strongest predictor of death, followed by BMI and age. In those with better eGFR and better pre-operative creatinine levels, age (*≥* 77.5 years) was the next discriminating factor, followed by the presence of type II diabetes.

**Fig. 4:**
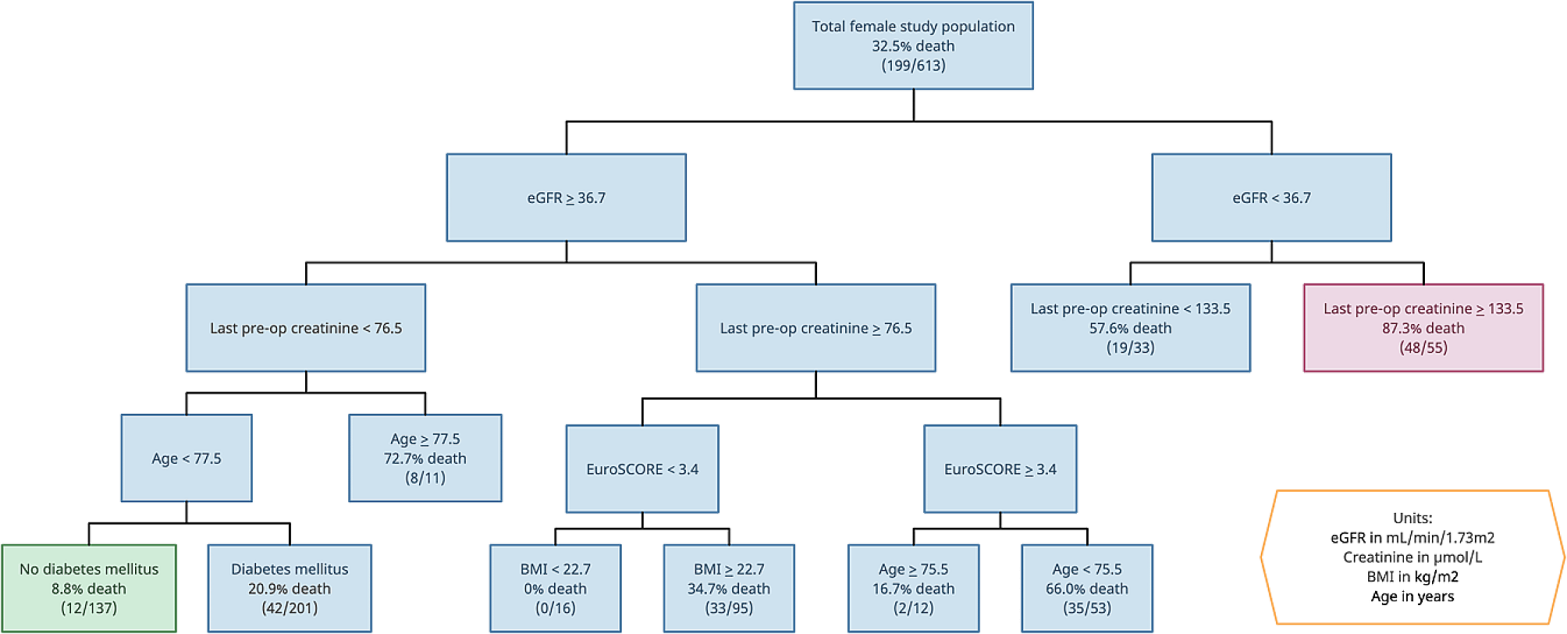
CART analysis for mortality after CABG in females. Variables branching off to the right are associated with higher mortality. Variables branching off to the left are associated with lower mortality. Green: combination with the best prognosis. Red: combination with the worst prognosis

**Fig. 5:**
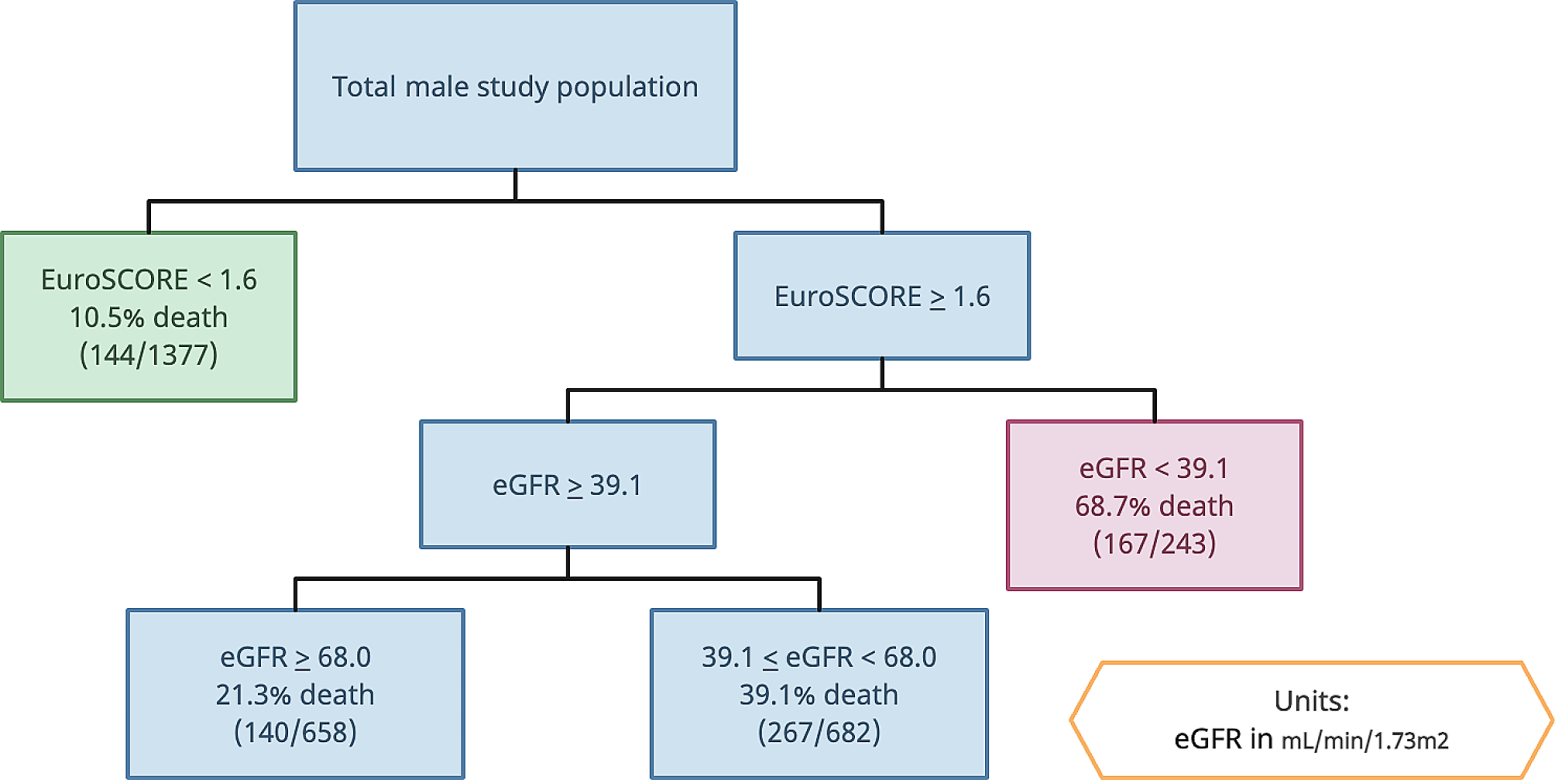
CART analysis for mortality after CABG in males. Variables branching off to the right are associated with higher mortality. Variables branching off to the left are associated with lower mortality. Green: combination with the best prognosis. Red: combination with the worst prognosis

For CART analysis results specific to male patients, the most important predictor of death was logistic EuroSCORE *≥* 1.6. In patients with higher EuroSCORE, eGFR was the next best predictor. This was divided into three groups with eGFR (mL/min/1.73m^2^), from worst to best prognosis, < 39.1, 39.1 (inclusive) to 68.0, and *≥* 68.0. The detailed cut-off values for each factor and the rate of death for each combination of risk factors can be seen in Figs. 4 and 5 for females and males respectively.

## Discussion

### Mortality rate

In this single center study, total mortality rate after isolated CABG over a maximum of 14.7 years follow-up was 25.7% (32.5% for females, 24.3% for males). The 30-day, 1-year, 5-year, 10-year and 14.5-year overall mortality rates were 1.5%, 2.9%, 9.9%, 22.8% and 36.3% respectively. Short-term mortality for CABG is often defined as death within the same hospital admission or within 30 days [3]. Short-term mortality has been commonly used to determine hospital performance and quality for cardiac surgery [9, 10]. For comparison, the ASCERT study on close to 350,000 isolated CABG patients reported an estimated mortality of 3.2% at 30 days and 8.1% at one year [11]. The New York State Cardiac Surgery Reporting System database saw 1-year, 5-year and 7-year mortality rates in patients who underwent isolated CABG to be 6.2%, 17.6% and 24.2% respectively [12]. A study on the Danish population reports 30-day, 1-year and 10-year mortality rates of 3.2%, 6.0% and 30.8% [13]. The multi-centre randomized controlled SYNTAX trial [14] and its 5-year [15] and 10-year [16] follow-ups reported post-CABG all-cause mortality rates of 3.5% at 1-year, 11.4% at 5-years and 24% at 10-years. There remain few large studies of CABG long-term mortality at 10 years and beyond. A comparison of mortality rates between these studies and those observed in our institution is summarised in Table 5. Mortality rates at our institution over the study period appear to be lower at all timepoints of follow-up compared to these other studies.

Nevertheless, the risk profile of patients undergoing CABG vary widely across different populations and institutions, which can inherently affect surgical outcomes. It would thus be inappropriate to assess the quality of care of an institution by comparing absolute mortality rates alone. Risk stratified data is essential for quality analysis, meaningful comparison of outcomes, and improvements of outcomes [17].

**Table 5:** Comparison between short- and long-term mortality rates observed in this study and those reported in various large studies. Observed institutional mortality rates were lower at all time points post-CABG

	All-cause Mortality Rate (%)				
Duration post-CABG	Observed	ASCERT Study^11^	New York State Cardiac Surgery Reporting System^12^	Danish Study^13^	SYNTAX (including follow-up studies)^14, 15, 16^
30 days	1.5	3.2	-	3.2	-
1 year	2.9	8.1	6.2	6.0	3.5
5 years	9.9	-	17.6	-	11.4
7 years	-	-	24.2	-	-
10 years	22.8	-	-	30.8	24
14.5 years	36.3	-	-	-	-

### Risk factors for CABG mortality

From univariate Cox analysis, the factors found to be predictors of mortality were: age, female gender, BMI, hypertension, diabetes mellitus, pre-operative renal failure on dialysis, COPD, higher last pre-operative creatinine level, lower eGFR, logistic EuroSCORE, heart failure, NYHA class II, III and IV, and LVEF. After adjustment for other risk factors, multivariate analysis showed that all these factors remained predictors of mortality, except for the female gender and logistic EuroSCORE. These risk factors have also been associated with increased mortality in a large number of other studies [3, 4].

Given that the outcomes of CABG are dependent on multiple variables, many algorithms have been developed to calculate a composite mortality risk score based on distinctive characteristics, co-morbidities and peri-operative features. Some of the most widely recognized and utilized algorithms are the EuroSCORE and the STS score [4–7]. Nilsson et al. compared 19 such algorithms in patients undergoing isolated CABG and found that EuroSCORE displayed the highest discriminatory power for both 30-day and 1-year mortality, followed by the New York State and the Cleveland Clinic algorithms [18]. Yet, these scoring systems were developed based on predominantly Western populations. It has been reported that EuroSCORE II overestimated the post-operative mortality in many recent studies conducted in Europe and Asia. The STS scoring system is also reported to be inaccurate in most Asian centers [19]. Asian populations undergoing CABG are known to differ in characteristics from Western populations, including having a younger age, and a higher percentage of diabetes and hypertension [19, 20]. Furthermore, both algorithms were designed to predict operative and short-term mortality, but not long-term mortality. Our study found that logistic EuroSCORE was not significantly associated with overall mortality after risk-adjustment.

Statins are an integral agent in cardiac care for both medical and post-surgical therapy [21, 22], suggesting that lipids play a role in the outcomes of cardiac surgery. However, we found that dyslipidemia did not significantly impact mortality. This has also been echoed by other studies [23]. The absence of lipid levels from the EuroSCORE and STS risk calculator for isolated CABG further suggest a lack of significant association between dyslipidemia and CABG mortality. An analysis of recent RCTs and meta-analyses show that pre-operative administration of statins did not appear to affect the incidence of cardiovascular events and overall mortality peri-operatively. However, they did reduce the risk of post-operative atrial fibrillation, and shortened hospital and ICU stay [24]. Another study reported that peri-operative administration of statins had a protective effect for all-cause and cardiovascular mortality, but that there was no association between lipid levels and mortality [25].

In another study on the Singapore population, Luo et al. [19] identified age, ethnicity, congestive heart failure, abnormal heart rhythm, aortic atherosclerosis, eGFR, peripheral vascular disease, critical preoperative status and emergency surgery as major factors correlated to mortality. The inclusion of these factors for analysis in future investigations can be considered for a more comprehensive identification of risk factors. We also recognise that the types of conduits utilized in CABG surgery significantly impact long-term survival benefits [26]. The type and number of conduits used were not included as risk factors in this study. However, all patients minimally received a left internal mammary artery graft.

### Use of CART analysis

CART analysis was employed to identify the strongest predictors of death and the cut-off values for the risk factors to stratify isolated CABG mortality risk. The appeal of CART analysis is the simplicity in which high-risk population can be predicted. CART models identify the relative importance of various clinical parameters without pre-specification of possible interactions [27, 28]. Regression techniques are employed to estimate the average effect of an independent variable on the probability of having a dependent variable, while accounting for other factors. Thus, CART analysis would not be used as a substitute for proven regression techniques in this type of situation. [27, 28]

Although multiple risk factors have been identified in the past, a clear prioritization for stratification of CABG patients at risk for death has not been established yet. CART analysis could demonstrate the factors which are particularly important in a model with regards to explanatory power and variance. Furthermore, it has advantages over more traditional methods, such as multivariate regression; it is inherently non-parametric and does not require a priori categorization of the data. [27, 28]

### Impact of renal function on mortality

The negative impact of poor renal function on CABG outcomes is well established [29–31]. Cooper et al. reported a mortality rate of 1.3% for patients with normal renal function, 9.3% for those with severe renal dysfunction not on dialysis, and 9.0% for those who were dialysis-dependent [29]. Gallagher et al. also reported that renal dysfunction increases 5-year adjusted mortality (HR 1.32; 95% CI 1.08–1.61). Both chronic kidney disease (CKD) and CAD increase risk and accelerate progression of the other disease [32, 33], thus many patients undergoing CABG also have impaired renal function. This risk factor of renal impairment plays an even bigger role as shown from our study, as Asians have an increased risk for renal disease compared to Whites [34]. Singapore also has a higher prevalence of CKD at 15.6%, compared to 13.1% in the USA, 10.2% in Europe and 13.7% in Korea [34].

CART analysis showed that for overall mortality after CABG, the strongest predictor of death was pre-operative eGFR. The cut-offs that stratify patients into different risks groups were eGFR < 46.9mL/min/1.73m^2^, 46.9 *≤* eGFR < 77.8mL/min/1.73m^2^, and eGFR *≥* 77.8mL/min/1.73m^2^. In females, the most important risk factor is also eGFR, whereas in males it is the second most important risk factor after logistic EuroSCORE. Grossly translating this into KDIGO CKD staging, this suggests that patients with CKD stage 3B (defined as eGFR 30-44mL/min/1.73m^2^) and above are at greatest risk of CABG mortality. Despite this, a meta-analysis [35] of outcomes of PCI vs. CABG in patients with CKD reported that CABG remains superior to PCI in terms of mortality and major adverse cardiovascular or cerebrovascular events. This suggests that CABG may be the preferred re-vascularization strategy for patients with concomitant CAD and CKD. Naturally, one would infer that with more end-organ damage, mortality would be expectantly higher, and this is shown from this study.

### Impact of gender on mortality

A higher mortality in females who have undergone CABG compared to males has been well-documented in many studies [13, 36–38]. In our study, females had a higher rate of absolute mortality compared to males (HR 1.42 [95% CI (1.22–1.67)], *p* < 0.001) before risk adjustment through multivariate analysis. However, while there is common consensus on the increased unadjusted mortality in females, existing literature is divided when using multivariate analysis. Some studies have found no difference in mortality between both genders after adjusting for risk factors [39], similar to our study results. Others report an increased risk of mortality in females even after adjustment [36, 38, 40].

Poorer outcomes in females, independent of other risk factors, have been attributed to smaller vessel diameter, underutilization of the internal mammary artery, and hormonal differences [38]. Females have a smaller luminal diameter of coronary arteries, even after risk adjustment for body size, which leads to increased thrombogenicity [38, 41]. The smaller vessel size also increases the technical difficulty of the surgery. Another postulation is that a difference in hormonal signaling involving estrogen, progesterone and testosterone receptors lead to a difference in vascular relaxation between the two genders, although this mechanism of action remains unclear [41].

This may also be due to females having poorer baseline characteristics at the time of undergoing CABG. In line with this, we found no increased risk of mortality in females after adjustment for risk factors. In our study, the female population was older than the male population. The females subjects had a greater incidence of hypertension and diabetes mellitus, as well as lower renal function and LVEF than the male subjects. This is in keeping with other studies done across different decades and populations [36, 39]. Females tend to present and be referred for CABG at an older age. With advancing age, they often have more significant co-morbidities like diabetes, hypercholesterolemia, hypertension, and cerebrovascular disease, all of which may contribute to increased CAD severity. Jawitz [42] asserts that there exist clear sex disparities in the management of CAD patients from diagnosis to referral for treatment and surgical approaches used. Other studies also found delayed intervention in females due to more atypical symptoms of myocardial infarction [38]. The identification of modifiable risk factors from the non-modifiable intrinsic risks in the female gender can be important to guide changes in clinical practices to improve outcomes of CABG in females.

### Study limitations

There are several limitations to this study. Firstly, we recognise a possible selection bias as a large proportion of the population that underwent isolated CABG at our institution was excluded due to missing data. Of the 6,992 patients who underwent isolated CABG over the study period, 3,372 had missing NYHA data, 17 had missing LVEF data and 30 had both missing NYHA and LVEF data – resulting in a total of 3,419 patients with incomplete dataset. The reason for this missing data could be largely attributed to a migration to a new database used by our institution in 2013. Nevertheless, the decision was made to include only patients with complete dataset given that both NYHA and LVEF have been shown to significantly affect mortality. To address the issue of selection bias, we thus analysed the excluded cohort separately *(see Supplementary Material)*, which has shown that even in this group, renal function quantified using eGFR remains the strongest predictor of long-term post-CABG mortality.

Next, the results may not be generalizable to patients undergoing CABG concurrently with other cardiac surgical procedures such as valve replacements, as only patients who underwent isolated CABG were included. Additionally, the retrospective nature of it leads to confounding factors. This study also looked solely at mortality rates and did not include other post-operative endpoints such as myocardial infarction, need for repeat revascularization, stroke and renal failure, which collectively determine the outcomes and degree of benefit of the procedure.

The logistic EuroSCORE was used for analysis despite the transition to the updated EuroSCORE II in 2012. This was due to our study duration spanning from 2008 to 2017, crossing the transition period. Thus, only the logistic EuroSCORE was available for patients who underwent CABG prior to 2012.

## Conclusion

To the best of our knowledge, this is the largest study on the Southeast Asian population to determine the relative importance and interactions of clinical parameters for risk of death late after CABG. Amongst the risk factors, we concluded that poorer renal function, quantified by lower eGFR, has the strongest discriminating power in predicting mortality. Patients with CKD stage 3B and above are at highest risk for post-CABG mortality. Amongst the other risk factors, logistic EuroSCORE, older age, diabetes and BMI had a relatively greater impact on mortality. Surprisingly, the female gender is not amongst the top few risk factors for death on CART analysis despite a stark divergence in mortality between genders since the early post-operative period. This demonstrates the value of combining variables to identify clinically meaningful sub-groups that are at highest or lowest risk for death after CABG. We hope our study findings highlight the importance of optimizing current medical therapy to preserve renal function upon the diagnosis of any atherosclerotic disease or risk factor contributing to coronary artery disease.

### Electronic supplementary material

Below is the link to the electronic supplementary material.

Supplementary Material 1

## Data Availability

No datasets were generated or analysed during the current study.
