# Supplementary material for: Long-term mortality after isolated coronary artery bypass grafting and risk factors for mortality
Source: J Cardiothorac Surg. 2024 Jul 10;19:429. doi: 10.1186/s13019-024-02943-0 (PMC11234617; doi:10.1186/s13019-024-02943-0)

**SUPPLEMENTARY MATERIAL**

Of the 6,992 patients who underwent isolated CABG over the study period, 3,372 had missing NYHA data, 17 had missing LVEF data and 30 had both missing NYHA and LVEF data – resulting in a total of 3,419 patients with incomplete dataset. In a comparison of risk factors present in the included versus the excluded populations (Table 1), there is a significant difference in dyslipidemia, diabetes mellitus, pre-operative renal failure on dialysis, last pre-operative creatinine level and eGFR. Nevertheless, the percentage of these two groups with dyslipidemia (82.8% vs 80.2%) and diabetes mellitus (51.7% vs 48.0%) are observed to be similar. The included cohort has nearly twice the percentage of patients with pre-operative renal failure on dialysis (4.3% vs 2.6%). The included population has a lower median eGFR (71.4 vs 74.7) and higher last pre-operative creatinine (88 vs 85), although the median values are once again similar. In conclusion, the final group included in the study has lower renal health as characterised by lower eGFR and higher pre-operative creatinine, however the median values of these risk factors are similar in both groups.

CART analysis was applied to all patients with available LVEF data (N= 6,945) (Figure 1) with NYHA removed from the risk factors. eGFR, at a cut-off of <47.0, remains the strongest predictor of death. Even when analysing the excluded population alone (N=3,419) (Figure 2), eGFR, at a cut-off of <50.1, is once again the strong predictor. However, in both of these groups, a further stratification into patients with eGFR <22.8 have the overall worst survival rate.

We hence conclude that renal function, quantified using eGFR, is the strongest predictor of long-term post-CABG mortality.

**Table 1. Comparison of risk factors observed in excluded versus included population.**

Continuous variables are expressed as median (IQR), and categorical variables as n (%).

|  | **Included population (N=3,573)** | **Excluded population (missing NYHA and/or LVEF) (N=3,419)** | **p-value (Mann–Whitney test/Chi-square test)** |
| --- | --- | --- | --- |
| **Age (years)** | 62 (55-68) | 62 (55-67) | 0.390 |
| **Female gender** | 613 (17.2%) | 536 (15.7%) | 0.095 |
| **Body mass index (kg/m^2^)** | 25.1 (22.9-27.8) | 25.0 (22.8-27.7) | 0.180 |
| **Hypertension** | 2,694 (75.4%) | 2,551 (74.6%) | 0.450 |
| **Dyslipidemia** | 2,958 (82.8%) | 2,742 (80.2%) | 0.005 |
| **Diabetes mellitus** | 1,846 (51.7%) | 1,642 (48.0%) | 0.002 |
| **Pre-operative renal failure on dialysis** |  |  | <0.001 |
| No | 3,419 (95.7%) | 3,329 (97.4%) |  |
| Yes | 154 (4.3%) | 90 (2.6%) |  |
| **COPD** | 34 (1.0%) | 31 (0.9%) | 0.850 |
| **Last pre-operative creatinine level (μmol/L)** | 88 (74-105) | 85 (72-101) | <0.001 |
| **eGFR (mL/min/1.73m^2^)** | 71.4 (54.9-91.1) | 74.7 (58.2-93.3) | <0.001 |
| **Logistic EuroSCORE** | 2.1 (1.3-3.3) | 2.0 (1.3-3.3) | 0.180 |
| **Heart failure** | 219 (6.1%) | 206 (6.0%) | 0.860 |

**Figure 1. CART analysis of all patients who underwent isolated CABG during the study period with available LVEF data.** Variables branching off to the right are associated with higher mortality. Variables branching off to the left are associated with lower mortality. Green: combination with the best prognosis. Red: combination with the worst prognosis.


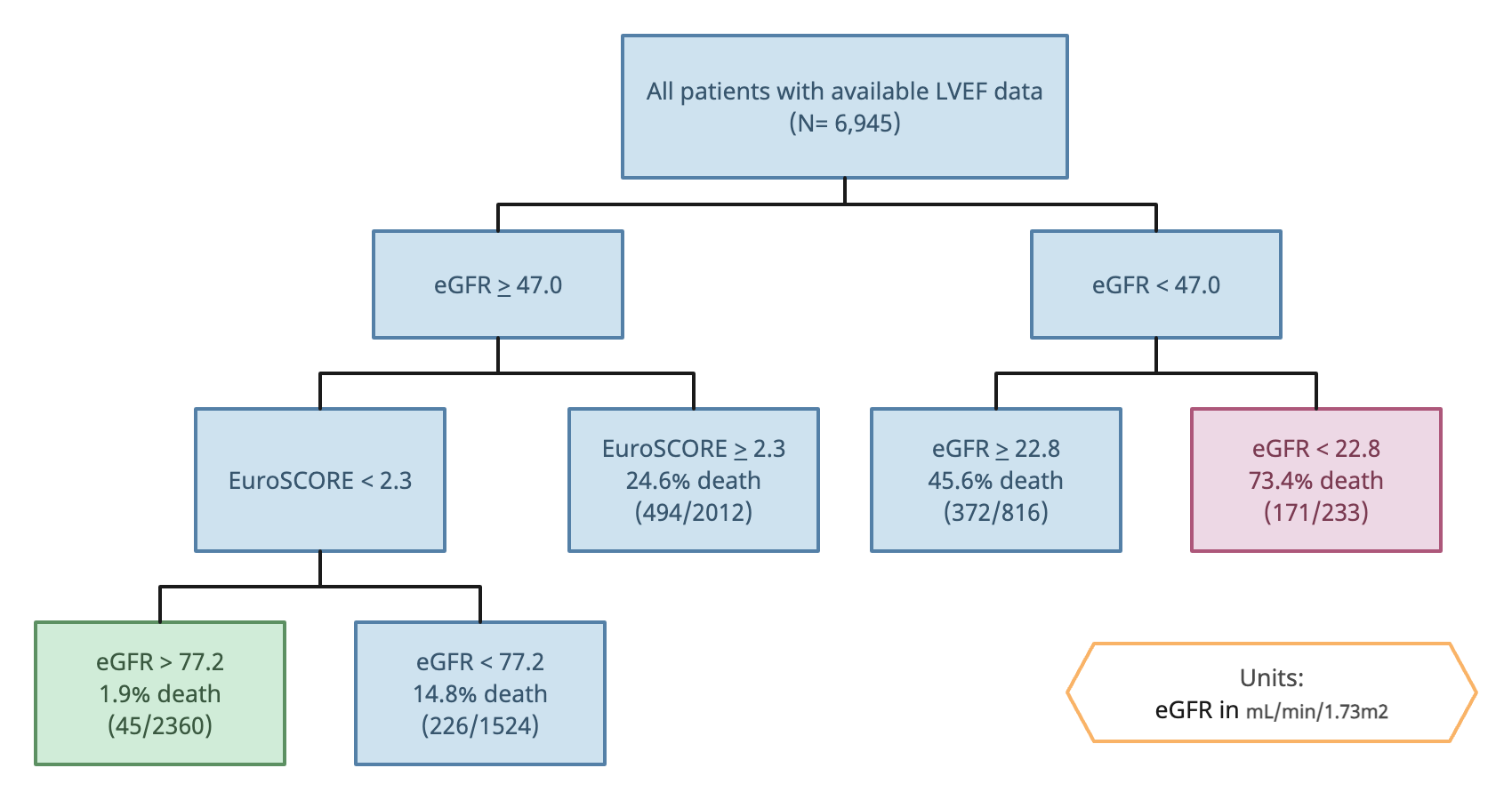


**Figure 2. CART analysis of patients who were excluded from the study due to missing LVEF or NYHA data.** Variables branching off to the right are associated with higher mortality. Variables branching off to the left are associated with lower mortality. Green: combination with the best prognosis. Red: combination with the worst prognosis.


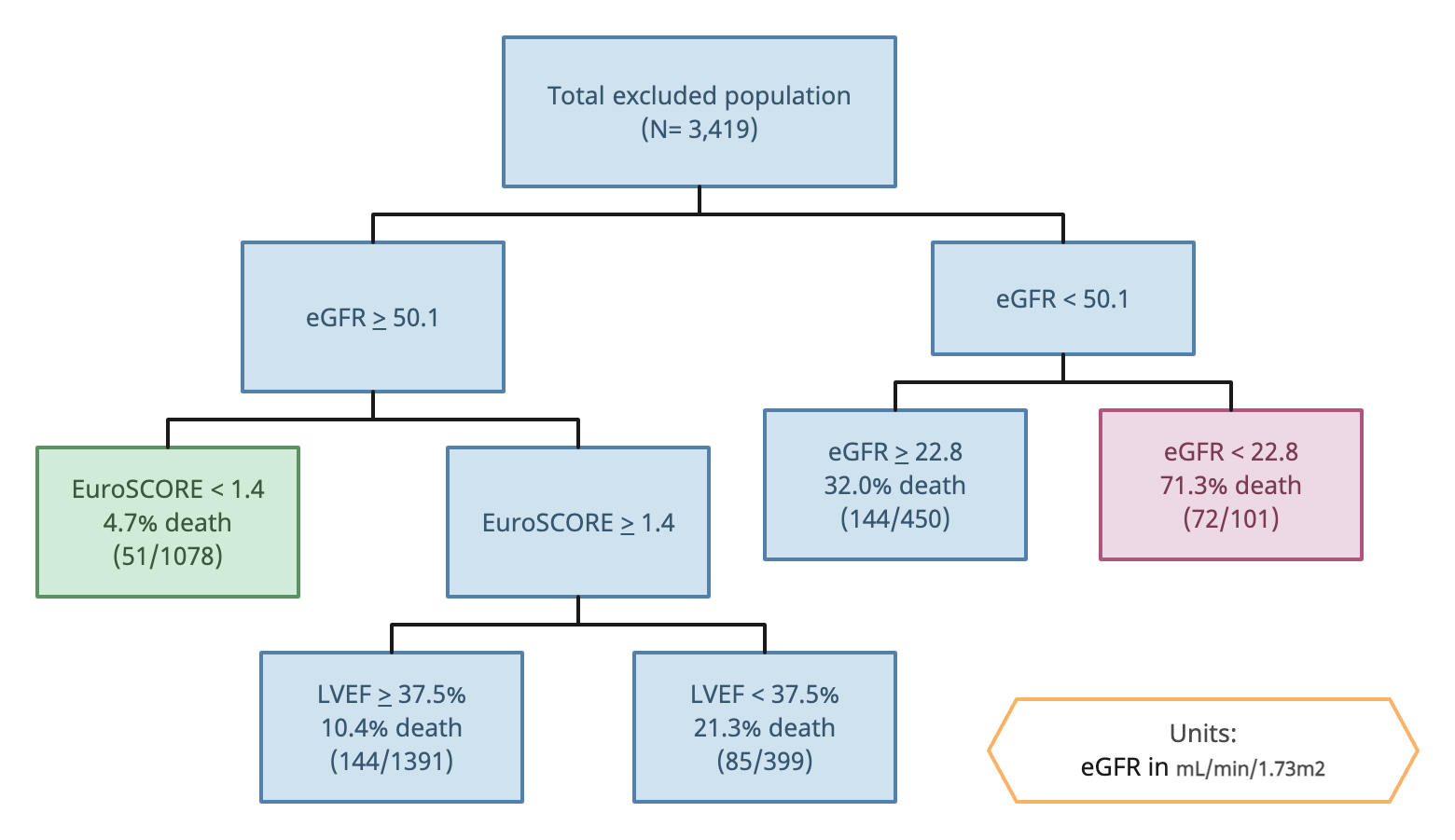

Supplement: Supplementary file 1 — Supplementary Material 1 [file 13019_2024_2943_MOESM1_ESM.docx]
